# The dentist’s operating posture – ergonomic aspects

**Published:** 2014-06-25

**Authors:** C Pîrvu, I Pătraşcu, D Pîrvu, C Ionescu

**Affiliations:** * Department of Oro-Dental Diagnosis, Ergonomics and Research Methodology, "Carol Davila" University of Medicine and Pharmacy, Bucharest; ** Department of Dentures Technology and Dental Materials, "Carol Davila" University of Medicine and Pharmacy, Bucharest

**Keywords:** balanced posture, unbalanced posture, working positions

## Abstract

Abstract

The practice of dentistry involves laborious high finesse dental preparations, precision and control in executions that require a particular attention, concentration and patience of the dentist and finally the dentist’s physical and mental resistance. The optimal therapeutic approach and the success of practice involve special working conditions for the dentist and his team in an ergonomic environment. The meaning of the posture in ergonomics is the manner in which different parts of the body are located and thus the reports are established between them in order to allow a special task execution. This article discusses the posture adopted by dentists when they work, beginning with the balanced posture and going to different variants of posture. The ideal posture of a dentist gives him, on the one hand the optimal working conditions (access, visibility and control in the mouth) and on the other hand, physical and psychological comfort throughout the execution of the clinical acts. Although the theme of dentist posture is treated with great care and often presented in the undergraduate courses and the continuing education courses on ergonomics in dentistry, many dentists do not know the subject well enough nor the theoretical issues and therefore nor the practical applicability. The risk and perspective of the musculoskeletal disorders related to unbalanced postures should determine the dentists take postural corrective actions and compensation measures in order to limit the negative effects of working in a bad posture.

## Introduction

The practice of dentistry involves laborious high finesse dental preparations, precision and control in executions that require a particular attention, concentration and patience of the dentist and finally the dentist’s physical and mental resistance. The optimal therapeutic approach and the success of practice involve special working conditions for the dentist and his team in an ergonomic environment. The ergonomics applied in dentistry focuses on the practitioner and his team as the most important resource of the practice.

 In the field of ergonomics applied in dentistry one of the most discussed theme is the working posture of the dentist. The special attention on this topic is explained by the widely recognized and accepted fact that posture is the key of preventing the musculoskeletal disorders.

 The meaning of the posture in ergonomics is the manner in which different parts of the body are located and thus the reports established between them in order to allow a special task execution. In dentistry, the working position represented by the spatial arrangement of the dentist’s entire body around the patient must be distinguished. This differentiation is useful to understand the working conditions.

 The ideal posture of a dentist gives him on the one hand, optimal working conditions (access, visibility and control in the mouth) and on the other hand, physical and psychological comfort throughout the execution of the clinical acts. A "good" posture provides the dentist more working energy, a reduced stress level, increased comfort, lack of pain and muscular tension and a lower risk for therapeutical errors. A "bad" posture induces premature fatigue, pain, stress and a negative attitude to work, high-risk for musculoskeletal disorders and a poor quality of work [**1**].

 This article discusses the posture adopted by dentists when they work, beginning with the balanced posture and going to different variants of posture. The balanced posture is a posture that should be a reference point for the postures that are daily adopted by dentists. Literature provides numerous data on this topic, many authors being concerned about it. The instances in which the dentists severely deviate from an ergonomic working style (which implies a correct posture) are extremely numerous from many reasons. The working posture of the dentist is strongly influenced by the relationship between his body and the different elements of his workstation, so that an incorrectly designed workstation and/or incorrectly used workstation affect the posture. By workstation, we understand all the spatial and environmental elements with which the dentist interacts while working [**2**].

 Among the spatial elements of the dentist’s workstation the following can be identified: the dentist’s stool, the patient lying in the anatomical chair, the components of the dental unit (the dentist’s cart, the lamp, the vacuum system and the control pedal), the fixed elements surrounding the working area (the fixed furniture, the mobile case and all the tools and equipment placed on it), the equipment used and the presence of the dental assistant and the working relationship established with her. The multitude of these items reflect the extent to which the dentist can be conditioned and limited at his workstation, which may lead to an increased physical and mental stress, often manifested by a feeling of "frustration" experienced during all the working stages.

 The dentist’s posture evolved from the orthostatic posture to a seated posture especially on the account of adopting the four hands working style [**1**]. Today, we meet the two posture possibilities (the orthostatic posture and the seated posture) used in varying degrees. In practice, we often observe that many dentists work in incorrect postures because of their habitude, working routine and poorly designed workstations. All these conditions act on the lack of the dentist’s information in the field of ergonomics and on ignoring the fact that the human body has its adaptive limits. Beyond these limits, the dentists are exposed to the risk of professional illness.

 The posture described in "ISO Standard 11226 Ergonomics - Evaluations of static operating postures" is recommended for the dentists and is called balanced or neutral posture. The balanced or neutral posture is a reference point for the correct working posture and it is recommended to be maintained within the limits imposed by the practice conditions, throughout all the stages of the clinical acts. This is a seated posture - natural, unforced, stress free and symmetrical - that takes into account the loco-motor physiology of the human body. The neutral posture is the result of the general ergonomic studies adapted to the needs of the dental practice. This posture is comfortable (assuming minimal contractions and muscular tensions), stable (stabilizing all body segments and joints) and symmetrical – the defining element for postural balance. The notion of "neutral" derives from the fact that each joint of the body has a neutral zone to which the movements are reported and which recurrent exceeding could generate the joint and muscles overstrain [**1**]. The balanced or neutral posture is the result of a complex positioning of the different body segments, each of them with its neutral limits for a risk-free use [**3**].

 The balanced posture features can be summarized as it follows [**4**]:

 -a straight back and respect for the body symmetry; avoiding rounding the back into "C" shape;

 -forward inclination of the trunk of a maximum of 20°; a greater forward inclination, the tilting to a side and the trunk rotation are contraindicated;

 -forward inclination of the head up to 20-25° from the trunk;

 -the arms placed along the body, forward oriented within 10°; the forearms raised up to 25° from the horizontal line;

 -the angle between the thighs and shanks of 105-110° or more;

 -the thighs apart up to 45°, avoiding a rigid fixation of the hip joint;

 -the shanks oriented perpendicular to the floor or slightly posterior;

 -the feet on the floor oriented forwards in the same plane with the shanks; when the feet are symmetrically positioned below the operator hands, the posture is balanced.

 The postural symmetry implies all the body horizontal lines (the eyes, shoulders, elbows, hips and knees horizontals) being parallel and perpendicular to the median line of the body [**4**].

**Fig. 1:**
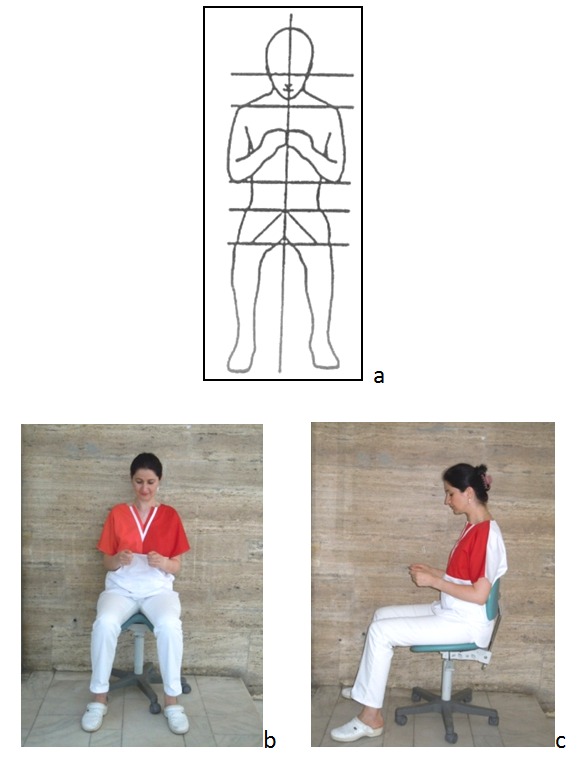
a,b,c The balanced posture

 Preserving the balanced posture and its symmetry throughout the clinical act is largely conditioned by the relationship established between the dentist and the intraoral working field. In an ideal situation, the surface of the treated teeth should be parallel to the front of the dentist and his view oriented perpendicular to the working field. It is recommended that the distance between the working field and the dentist’s eyes is of 35-40 cm or slightly higher for very tall dentists. When this relationship is not established or it is lost during the clinical act, the dentist’s eyes will look for it and the dentist will depart instinctively from the balanced posture. To establish such a relationship it is important to pay attention to the dentist’s position around the patient and the patient’s head position on the headrest. To position the patient’s head it is important to use all his possibilities of motion: extension and flexion, rotation right or left and side flexion right or left in varying degrees and combinations [**5**].

 The balanced posture does not require a rigid body attitude. The dentist has the freedom to move within some limits so that no harmful positions result. Gentle rebalancing movements are often made. The specialty literature uses the concept of active and passive balanced posture. The active balanced posture involves the dentist supporting the back straight (the physiological spine curvatures and the "S" spine form) only by means of paravertebral muscles tonicity. The passive balanced posture is characterized by the use of lumbar support provided by the seat back [**4**].

 To maintain an ideal posture for as long as possible, the working equipment and the working space (the workstation) must be "tailored" to the dentist and it must fit him like a glove. This ideal situation corresponds to the most favorable positioning of the dentist’s head, body segments, dominant hand and fingers, elements that can be tested by proprioceptive control according to the proprioceptive derivation concept. Numerous tests have been done on the dentists who were left to look for blindly and to feel the most convenient working body position (posture) that allowed them a high working finesse and a good manual control, asking them to try to ignore their equipment around and their old habits. The results showed a number of preferences: placing the dominant hand index around the armpit level on the midline of the body and the dentist sitting with the back straight without using support from the backrest [**6**]. In order to take and use the medical tools, the dentist extends his arm horizontally and vertically so that allowing this service without compromising the balanced posture, the tools must properly positioned [**6**]. 

 Using the magnification systems (glasses and corrective lenses, loupes and telescopes, the operating microscope) deserves a special attention because it could have serious implications for posture. Properly chosen and adjusted, the magnification systems can prevent the bending of the dentist’s head and the development of the musculoskeletal disorders. On the other hand, their misuse could have adverse effects, increasing the risk of musculoskeletal injuries or aggravating them [**7**].

 The prolonged static postures are strongly incriminated in the etiology of musculoskeletal disorders and therefore they deserve a special attention. Sitting on the chair for a long time involves the activation of paravertebral muscles and a substantial "charging" of the lumbar curvature of spine higher than standing (2.7). To maintain a posture for a long time, including a balanced posture, predominantly involves a static muscular effort. The static work is more tiring than the dynamic work though it requires lower oxygen consumption. In a static work to the nerve centers of the cerebral cortex, a large number of impulses from proprioceptors of muscles and tendons reach the same place, this meaning a continuous long time excitation of these cerebral cortex nerve centers. This mechanism explains why the static work is more tiring than the dynamic work [**8**]. On the other hand, in a static posture (especially if it is incorrect) the oxygen supply to the muscles may be affected, which translates quickly through muscle inefficiency and even pain. In the dynamic work functions, the muscle pump (the alternative contraction and relaxation of muscles) promotes blood circulation [4,9-11]. Studies have been made which showed that maintaining the posture requires more than 50% of the body muscles to be isometrically contracted, which means an intense muscular effort. This effort can lead to muscular overworking, poor blood irrigation and an increased pressure on joints and muscles [**12**].

 When a prolonged static posture is added, other postures constraints its effects. On the other hand, the discomfort caused by a prolonged static posture and the required recovery times are directly proportional to the time spent in this manner [**13**].

 In order to compensate the effects of a static work and postural demands, many authors recommend a dynamic way of working. This includes:

 - employing the active balanced posture and the passive balanced posture alternatively;

 -using the short breaks between the patients in order to leave the stool and walk;

 -a working program with long demanding treatment sessions alternating to short and easier ones;

 -performing simple exercises between patients at the end of the program [**1**].

 Although the importance of the balanced posture to avoid prolonged static posture is widely recognized, some authors recommend the use of several alternative postures in order to employ more muscular groups [14-16]. A specific study shows that the dentists who work exclusively seated, suffer more pronounced pain in the lumbar curvature of spine compared with the dentists who alternate seated posture with the orthostatic posture [**17**]. There are authors who recommend changing the posture and position after maximum one hour of working. On the other hand, during the long clinical acts, a pause of five minutes after one hour of activity would be welcomed in terms of postural challenge to both the dentist and the patient. The position of the patient (especially head positions) may also become tiring when the clinical act is extended [**18**].

 The mental and psycho-emotional stress caused by the difficulty of working can induce an increased muscular tension, a reduced attention to posture and finally a wider body response to a bad posture [**7,10**].

 Using an ergonomic stool and adjusting it correctly and in an easy way has a major contribution in the adoption and maintenance of a balanced posture. Through its features, the dentist’s stool has to encourage a good posture [**2**].

 The stool’s height adjustment is necessary for the correct orientation of the thighs to the floor and a minimum angle of 105-110º between the thighs and the shanks. A too high position of the stool requires sitting on the edge, losing the weight distribution on the stool axis and losing the contact of the dentist’s back with the stool’s back. This way, the risk of slipping off the stool appears. When the stool is too low, the lumbar curvature of the spine is reduced through posterior rotation of the pelvis [**7**].

 The orientation of the dentist’s stool base can be horizontally or forwardly inclined. A horizontal stool base may cause a posterior rotation of the pelvis and reduction of the lumbar curvature of the spine while its forward inclination prevents this phenomenon. In addition, a horizontal stool base with a too large surface and a hard edge can generate a posterior compression of the thighs, which affects the shanks and legs blood supply. By tilting the stool base, this phenomenon is avoided. The tilting degree must be reduced at 5 to 15º because a bigger degree could determine sliding off the chair. A saddle-shaped stool base does not pose such problems, being favorable from this point of view. Both the forward inclination of the base and the saddle-shaped base conformation allow a trunk-thigh angle greater than 90° and a closeness to the patient which facilitates a better access and visibility in the patient’s mouth [**1,7**].

 Supporting the back on the backrest of the stool is necessary in order to avoid the muscular fatigue and reduction of the lumbar curvature of spine during long clinical acts. A properly support does not require a great height of the backrest, 20 cm being sufficient [**7**].The lumbar support is recommended to be set on the upper half of the lower back, where switching to thoracic convexity [**1**]. The relationship between the position and the stool base and the position of the backrest is especially important to reduce the lumbar curvature tension [**2**]. Increasing the angle between the torso and the thighs in order to position closer to the patient, has a favorable effect reducing the lumbar kyphosis [**13**].

 For the long lasting clinical acts is also important to support the arms on the special supports of the stool. This support addresses in the same time to the shoulders, preventing the back pain and the shoulder and neck tension that may occur due to the prolonged muscular tension. The stool with supports for arms is bigger and it seems to limit the free movements (especially if we do not have proper conditions to approach the patient). For this reason, it is often avoided by practitioners, but after a while of use and adaptation, the benefits become clear [**19**]. In order to respect the symmetry of a balanced posture it is recommended to use the supports for both arms simultaneously [**5**].

 The deviations from the balanced posture

 In practice, it is almost impossible for the dentists to maintain a balanced posture throughout a long clinical act even when this posture is very well known and initially adopted. Fully aware of the effects of an incorrect posture, the dentists should consider the deviations as rare as possible, of small amplitude and short duration. The wider and longer these deviations are, the more the risk of MSDs increases [**3**].

 The deviations from the balanced posture during the clinical act may be caused by:

 -an incorrect positioning of dentist around the patient according the working area;

 -an inadequate working level (often the mouth is too low);

 -an incorrect positioning of the patient's head (not rotated, tilted or extended enough);

 -avoiding to work in an indirect vision (working in the mirror is more difficult and requires extra effort fixing the image);

 As means of deviation from the balanced position, the following can be seen most often [1,12]:

 -the excessive bending of the dentist’s head and the extent of neck, the rotation and the tilting of the head;

 -the tilting and the rotation of the trunk on one side;

 -lifted arms (dominant, non-dominant or both) without adequate support on the trunk or on the arm support of the stool;

 -lifting one shoulder or both;

 -an increased thoracic curvature of the lumbar curvature reduction;

 -the angle between thighs and shanks below 90°.

 Avoiding the patient seat backrest and headrest margins, in order to gain access and visibility in the mouth, the patient’s shoulder or some tools create conditions for a poor unbalanced posture. The direct consequences of these "interferences" are twisting and tilting the body segments (head, shoulders and trunk) on one side and raising the elbows and shoulders. In the absence of a specific attention to the postural changes, these may become repetitive and of long standing. On the other hand, due to interferences, the need for a repetitive repositioning of the tools, the equipment or the patient, reduce the dentist’s chance of working in a balanced posture. The act of repositioning could compromise the posture favoring the occurrence of musculoskeletal disorders, reducing the access to the mouth and creating conditions for working incidents and poor lighting of the surgical field. All these conditions lead to physical and mental fatigue and tension. Avoiding interferences and the repetitive repositioning cause fatigue, confusion and conditions for musculoskeletal disorders [**6**].

 The frequent unidirectional trunk twisting and tilting is a consequence of a faulty positioning of the tools, equipment and materials. Positioning these useful items at a big distance outside the working area of the dentist (the circular area around the dentist described by the forearms length in his sight) forces him to deviate from the balanced posture. The help of the dental assistant regarding the tools also handing has a major influence on postural deviations of the dentist [**14**].

**Fig. 2:**
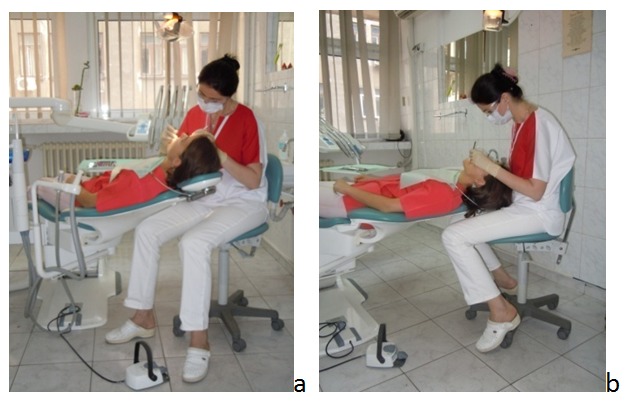
a,b The frequent deviations from the balanced posture

 The unbalanced dentist’s postures

 In dentistry practice, only the orthostatic posture can be often seen despite its many disadvantages. The dentist’s orthostatic posture is an asymmetrical posture with the body support mainly on the right foot and the internal organ compression on the right side due to bending towards the patient. Only certain short clinical acts should be done in this posture (e.g.: the bite records, the impressions, some extractions, the aesthetic evaluations) [9,11]. 

 The possible causes of maintaining this posture are: the habitude, the changing resistance, the intensive working rhythm, the feeling of losing some freedom of movements once sitting on the stool and working unassisted or assisted only in part by the dental assistant (the dentist himself takes the tools, which requires moving free in the office space for easy access).

**Fig. 3:**
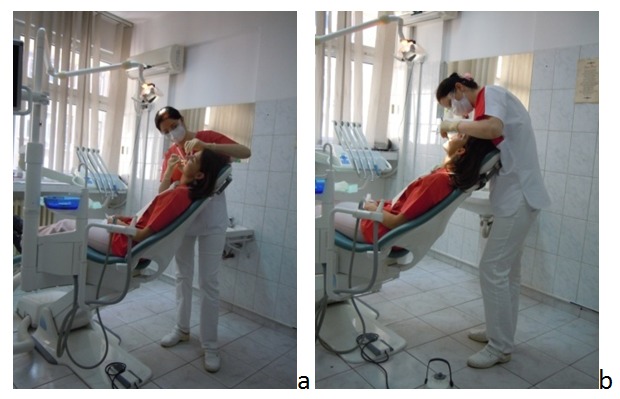
The orthostatic unbalanced posture

 Another dentist’s unbalanced posture is a seating posture with the upper body tilted on the right side caused by the vertical or only slightly oblique positioning of the patients. Compared with the orthostatic posture, it protects the shanks and legs but all the problems associated with of the marked long maintained asymmetry are still manifesting. Twisting the trunk in only one direction leads to serious muscular and joint unbalances and favors the low back pain [**20**]. 

**Fig. 4:**
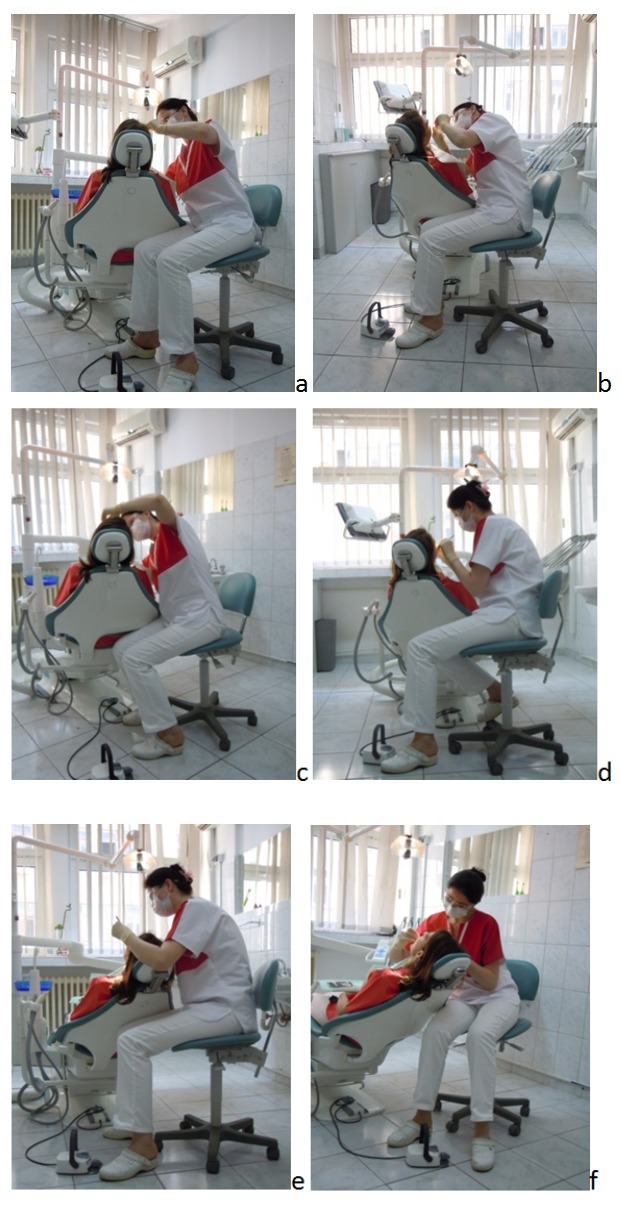
a-f The seated unbalanced posture

## Conclusions

Although the theme of the dentist’s posture is treated with great care and often presented in the undergraduate courses and the continuing education courses on ergonomics in dentistry, many dentists do not know the subject well enough, nor theoretically, and therefore nor its practical applicability. Unfortunately, many dentists are in the situation of feeling the negative effects of unbalanced postures in the first years of practice. In case musculoskeletal disorders arise, they should take correcting actions and compensatory measures in order to compensate the negative effects of the unbalanced posture. Each dentist who feels responsible for his health should reassess his working posture. A good posture is not a luxury and it does not require major investments but a rethinking of the way of working. The dentists should not live their professional life in terms of discomfort and musculoskeletal disorders perspective.
